# *Journal of Translational Medicine* reviewer acknowledgement 2013

**DOI:** 10.1186/1479-5876-12-23

**Published:** 2014-01-31

**Authors:** Francesco Marincola

**Affiliations:** 1 Sidra Medical and Research Center, Doha, Qatar

## Abstract

**Contributing reviewers:**

The editors of *Journal of Translational Medicine* would like to thank all our reviewers who have contributed to the journal in Volume 11 (2013).

## 

Katharine Abba

United Kingdom

Mario Abbud-Filho

Brazil

Ussama Abdel

United States of America

Alice Abdel Aleem

Qatar

Abhinav Acharya

United States of America

Raffaele Addeo

Italy

Daniel Aeschlimann

United Kingdom

Bharat Aggarwal

United States of America

Davide Agnello

France

Vincent Agnello

United States of America

Anurag Agrawal

India

Jing Ai

China

Pinar Akcakaya

Sweden

Mohammed Akhtar

United Kingdom

Nehad Alajez

Saudi Arabia

Salah-Eddin Al-Batran

Germany

Ramon Alemany

Spain

Luis F Alguacil

Spain

Radica Alicic

United States of America

Malcolm Alison

United Kingdom

Heike Allgayer

Germany

Fahd Al-Mulla

Kuwait

Cestmir Altaner

Slovakia

Rui Amaral Mendes

Portugal

Robert Amato

United States of America

She-Juan An

China

Yi Hua An

China

Helen Angell

France

Makoto Anraku

Japan

Alessandro Antonelli

Italy

Lionel Apetoh

France

Andrew Aplin

United States of America

Maria Virginia Appleyard

United Kingdom

Minoti Apte

Australia

Paolo Arese

Italy

Atsushi Aruga

Japan

Junichi Asaumi

Japan

Maria Libera Ascierto

United States of America

Paolo A. Ascierto

Italy

Vasilios Athyros

Greece

Joanne Attema

Australia

Michaela Aubele

Germany

Charles Auffray

France

Karen Avraham

Israel

Hammadi Ayadi

Tunisia

Yasu-Taka Azuma

Japan

Gunnar Baatrup

Denmark

Hideo Baba

Japan

Matthias Bache

Germany

Atul Bagul

United Kingdom

Li-Yuan Bai

Taiwan

Yu-Xian Bai

China

Mei Baker

United States of America

Michael Balls

United Kingdom

Andrea Balsari

Italy

Arun Bandyopadhyay

India

Gautam Banerjee

India

Rathindranath Baral

India

Jonathan Barratt

United Kingdom

Jirina Bartunkova

Czech Republic

Vinicius Bassaneze

Brazil

Analabha Basu

India

Davide Bedognetti

United States of America

Matteo Bellone

Italy

Helmut Beltraminelli

Switzerland

Andreas Bembenek

Germany

Fabian Benencia

United States of America

Angela Bentivegna

Italy

Carmen Berasain

Spain

Maria Ester Bernardo

Italy

Laia Bernet Vegue

Spain

Michel Bernier

United States of America

Cyril Berthet

France

Federico Bertuzzi

Italy

Levi Beverly

United States of America

Suk Ho Bhang

Korea, South

Dwaipayan Bharadwaj

India

Shephali Bhatnagar

United States of America

Nisan Bhattacharyya

United States of America

Xiu-Wu Bian

China

Zhaoxiang Bian

Hong Kong

Luigi Biancone

Italy

Ariane Biebl

Austria

Catherine Bisbal

France

Michael Bittner

United States of America

James Blanchette

United States of America

Martin Bluth

United States of America

Paul Bogner

United States of America

Margherita Bonamico

Italy

Martin Bonamino

Brazil

Roumiana Boneva

United States of America

Katherine Borden

Canada

Luciana Bordin

Italy

Jeffrey Borgia

United States of America

Emanuela Bostjancic

Slovenia

Pavel Bouchal

Czech Republic

Jason Bovenkerk

United States of America

James Bowen

Canada

Karim Brandt

Switzerland

Michel Braun

Belgium

Xandra Breakefield

United States of America

Patrick Breheny

United States of America

Ekua Brenu

Australia

Meredith Brisco

United States of America

Raf Brouns

Belgium

Lindsay Brown

Australia

Noor Np Buchholz

United Kingdom

Daniel Buergy

Germany

Abdelbaset Buhmeida

Saudi Arabia

Ronald Bukowski

United States of America

Kimberly Bullock

United States of America

Luigi Buonaguro

Italy

Dennis Burton

United States of America

Ludovico Buti

United Kingdom

Niels Henrik Buus

Denmark

Elif Cadirci

Turkey

Youqun Cai

China

Rodrigo Calado

Brazil

Alessandro Cannavo

United States of America

Martin Cannon

United States of America

Ya Cao

China

Concetta Castilletti

Italy

Justin Cates

United States of America

Christelle Cauffiez

France

Christophe Caux

France

Charles Cazanave

France

Giovanna Cenacchi

Italy

Simone Cenci

Italy

Gediminas Cepinskas

Canada

Laurence Chan

United States of America

Stephanie Chang

United States of America

Yu-Sun Chang

Taiwan

Caroline Chapman

United Kingdom

Mark Chariker

United States of America

Jaruek Charoensap

Thailand

Caifu Chen

United States of America

Chu-Huang Chen

United States of America

Chung-Ming Chen

Taiwan

Honglei Chen

China

Huei-Wen Chen

Taiwan

Jin-Qiang Chen

United States of America

Tianxiang Chen

China

Xi Chen

China

Yidong Chen

United States of America

Yih-Sharng Chen

Taiwan

Yong-Peng Chen

China

Zhe-Sheng Chen

United States of America

Pi-Wan Cheng

United States of America

Chandramu Chetty

United States of America

Harvinder Singh Chhabra

India

Maurizio Chiriva-Internati

United States of America

Sridar Chittur

United States of America

Bartosz Chmielowski

United States of America

Thinle Chodon

United States of America

Kyungmee Choi

Korea, South

Lotfi Chouchane

Qatar

Louis Chow

Hong Kong

I-Min Chu

Taiwan

Goh Eun Chung

Korea, South

Marcello Ciaccio

Italy

Gennaro Ciliberto

Italy

Caterina Cinti

Italy

Sara Civini

United States of America

Giovanni Cizza

United States of America

Francois X Claret

United States of America

Gillian Cockerill

United Kingdom

Matteo Coen

Switzerland

Alan Cohen

Canada

Peter Cohen

United States of America

Begonya Comin-Anduix

United States of America

Guillaume Counotte

Netherlands

Lisa Coussens

United States of America

Brendon Coventry

Australia

Francesca Crobu

Italy

Jianxiu Cui

China

Qinghua Cui

China

Fulvio D'acquisto

United Kingdom

Neetu Dahiya

United States of America

Marc Dahlke

Germany

Tina Dalianis

Sweden

Ashraf Dallol

Saudi Arabia

Deborah Damon

United States of America

Adrian Daneri-Navarro

Mexico

Alexandros Daponte

Greece

Silvia Darb-Esfahani

Germany

Ravi Dasu

United States of America

Jharna Datta

United States of America

Farshid Dayyani

United States of America

Jonathan D'cunha

United States of America

Piergiuseppe De Berardinis

Italy

Andrea De Maria

Italy

Valli De Re

Italy

Dominique De Seny

Belgium

Ayesha De Souza

United Kingdom

Nuria Del Olmo

Spain

Laurie Deleve

United States of America

Mario Delgado

Spain

Liane Deligdisch

United States of America

LF Dell'osso

United States of America

Lucia Gemma Delogu

Italy

Sandra Demaria

United States of America

Nerina Denaro

Italy

Jing-Yu Deng

China

Yuan Deng

China

Sebastien Deshayes

France

Angela Di Baldassarre

Italy

Massimo Di Maio

Italy

Catherine Diefenbach

United States of America

Marie-Caroline Dieu-Nosjean

France

Xiangwu Ding

China

Xiaoqiang Ding

China

Elena Dogliotti

Italy

Donald Doll

United States of America

Rosario Donato

Italy

Alena Donda

Switzerland

Hailong Dong

China

Zizheng Dong

United States of America

Frede Donskov

Denmark

Leonardo Dos Santos

Brazil

W. David Dotson

United States of America

Q Dou

United States of America

Paulo Dourado

Brazil

Caigan Du

Canada

Yujun Du

China

Chaojun Duan

China

Pradeep Dudeja

United States of America

Nicolas Dulphy

France

Jan Dutz

Canada

Kevin Dykstra

United States of America

Derek Dykxhoorn

United States of America

Laura Edsberg

United States of America

Hermann Eichler

Germany

Brian Eisner

United States of America

Daniel Eitzman

United States of America

Joanne Elena

United States of America

Ayman El-Kadi

Canada

Leisha Emens

United States of America

Ignacio Encío

Spain

Robby Engelmann

Germany

Kristina Eriksson

Sweden

Liao Eryuan

China

Helena Escuin

United States of America

Jim Fackler

United States of America

Areeg Faggad

Germany

Stefano Fais

Italy

Weiyi Fang

China

Cathy Sj Fann

Taiwan

Wendy Fantl

United States of America

Patrizia Farci

United States of America

Peiwen Fei

United States of America

Federica Felicetti

Italy

Jane Ferguson

United States of America

Mara Ferrandi

Italy

Pietro Manuel Ferraro

Italy

Clodoveo Ferri

Italy

Silvano Ferrini

Italy

Patrizia Ferroni

Italy

Seth Field

United States of America

William Figg

United States of America

Amy Firth

United States of America

Jens W Fischer

Germany

Paul Fisher

United States of America

Larry Fliegel

Canada

Caroline Ford

Australia

Marilena Formato

Italy

Albert Fornace

United States of America

Julien Fourcade

United States of America

Daniel Fowler

United States of America

Bernard Fox

United States of America

Claudio Fozza

Italy

Rodrigo Franco

United States of America

John Freiberger

United States of America

Satoshi Fujii

Japan

Daiju Fukuda

Japan

Tsuyoshi Fukushima

Japan

Niccola Funel

Italy

Mayuko Furuta

Japan

Alberto Fusi

Germany

Henrique De Azevedo Futuro Neto

Brazil

Giuseppe Gaipa

Italy

Massimiliano Galdiero

Italy

Maurizio Gallieni

Italy

James Galloway

United Kingdom

Bai Gang

China

Bin Gao

China

Rodrigo García-Baquero

Spain

Carol Gardner

United States of America

Andrei Gartel

United States of America

Wei Ge

China

Sarah Geisler

Switzerland

Anand Germanwala

United States of America

Jonathan Gershoni

Israel

Luca Giacomelli

Italy

Sandra Giest

Germany

Javier Gisbert

Spain

Annunziata Gloghini

Italy

Sacha Gnjatic

United States of America

Mary Goldring

United States of America

Nardhy Gomez-Lopez

United States of America

Yilei Gong

United States of America

Reginald Gorczynski

Canada

Norbert-Claude Gorin

France

Jones Graceli

United States of America

Marco Grasso

Italy

Johannes Graumann

Qatar

Quentin Grimal

France

Jochen Grommes

Germany

Nicole Gross

Switzerland

Johann Gudjonsson

United States of America

Cesare Guida

Italy

Farshid Guilak

United States of America

Junming Guo

China

Zhiqiang Guo

China

Zong Sheng Guo

United States of America

Ashish Gupta

India

Sanjay Gupta

United States of America

Katarzyna Guzinska-Ustymowicz

Poland

Mandana Haack-Sørensen

Denmark

Michael Hadjiargyrou

United States of America

Chariya Hahnvajanawong

Thailand

Roman Hajek

Czech Republic

Amira Hamzaoui

Tunisia

Bomie Han

United States of America

Saikh Haque

United States of America

Joshua Hare

United States of America

Damien Harkin

Australia

Curtis Harris

United States of America

Amanda Harvey

United Kingdom

Ryotaro Hashizume

Japan

Christos Hatzis

Greece

Thomas Hawke

Canada

Shoichi Hazama

Japan

Biao He

United States of America

Bulang He

Australia

John He

United States of America

Ping-An He

China

Sean Heffron

United States of America

Danielle Heideman

Netherlands

Peiman Hematti

United States of America

Ellen Henry

United States of America

Paul L Hermonat

United States of America

Helen Heslop

United States of America

Martin Hessner

United States of America

Falk Hiepe

Germany

Lucila Hinrichsen

Argentina

Catarina Hioe

United States of America

Sandra Hoegl

Germany

Melinda Hollingshead

United States of America

Bruce Hollis

United States of America

Martin Hoogduijn

Netherlands

Yuichi Hori

Japan

Sarah Hosgood

United Kingdom

Shirui Hou

United States of America

Christopher Hourigan

United States of America

Jillian Howlin

Sweden

Biliang Hu

United States of America

Guochang Hu

United States of America

Xi Chun Hu

China

Gonghua Huang

China

Peixin Huang

China

Derek Huffman

United States of America

Angela Bik-Yu Hui

Canada

Clifford Hume

United States of America

Keke Huo

China

Bonnie Hylander

United States of America

Ianko Iankov

United States of America

Thomas Ichim

United States of America

Juliana Idoyaga

United States of America

Giorgio Iervasi

Italy

Masahiro Imamura

Japan

Kimiko Ishiguro

United States of America

Ihsan Iskesen

Turkey

Ognen Ivanovski

Macedonia

N Gopalakrishna Iyer

Singapore

Lydie Izakovicova Holla

Czech Republic

Francesco Izzo

Italy

Jeffrey Jacobson

United States of America

Rune Jakobsen

Norway

Bahija Jallal

United States of America

Tae Jung Jang

Korea, South

Jelena Janjic

United States of America

Tavan Janvilisri

Thailand

Sebastien Jauliac

France

Tatiana Jazedje

Brazil

Jürgen Jenne

Germany

T-Y Jeon

Korea, South

Jihui Jia

China

Zhenyu Jia

United States of America

Chuanlu Jiang

China

Kaiyu Jiang

United States of America

Lili Jiang

China

Ling Jiang

China

Wen Jiang

United Kingdom

Chen Jihong

China

Kunlin Jin

United States of America

Ping Jin

United States of America

Young Ae Joe

Korea, South

Hanna Johnsson

United Kingdom

Bánóczy Jolán

Hungary

Jingfang Ju

United States of America

Achim Jungbluth

United States of America

Jussuf Kaifi

United States of America

Kenji Kakudo

Japan

Pawel Kalinski

United States of America

Bozena Kaminska

Poland

Hirohiko Kamiyama

Japan

Lana Kandalaft

United States of America

Chunsheng Kang

China

Jiuhong Kang

China

Ningling Kang

United States of America

Yubin Kang

United States of America

Sophia N Karagiannis

United Kingdom

Pratap Karki

United States of America

Mohammed Kashani-Sabet

United States of America

Masaru Katoh

Japan

Steven Katz

United States of America

Howard Kaufman

United States of America

Koji Kawakami

Japan

Yutaka Kawakami

Japan

Bhumsuk Keam

Korea, South

Bronya Keats

Australia

Benjamin Kefas

United States of America

Fergal Kelleher

Australia

Jennifer Kelly

United States of America

Candace Kerr

United States of America

Santosh Kesari

United States of America

Khandan Keyomarsi

United States of America

Amit Khera

United States of America

Paneez Khoury

United States of America

Wisam Khoury

Israel

Theo Kim

Germany

Stefan Kippenberger

Germany

John Kirkwood

United States of America

Rick Kittles

United States of America

Tohru Kiyono

Japan

Takako Kizaki

Japan

Lidija Klampfer

United States of America

Marijan Klarica

Croatia

Douglas Kniss

United States of America

Keith Knutson

United States of America

Stefan Kochanek

Germany

Kenichi Kohashi

Japan

Martina Kolackova

Czech Republic

Haruki Komatsu

Japan

Tadashi Kondo

Japan

Fumio Konishi

Japan

Chihaya Koriyama

Japan

Maciej Kosieradzki

Poland

Gary Koski

United States of America

Hervé Kovacic

France

Richard Koya

United States of America

Maarten Kraan

Sweden

Peter Krajcsi

Hungary

Jose E Krieger

Brazil

Smriti Krishna

Australia

Nikolaus Kroeger

Germany

Rebekka Krumbach

Germany

Yanan Kuang

Afghanistan

Selim Kuci

Germany

Helena Kuivaniemi

United States of America

Michael Kupferman

United States of America

Fin Kurreeman

Netherlands

Mamoru Kusaka

Japan

Kaoru Kusama

Japan

Sarah Kutscher

Germany

Taeg Kyu Kwon

Korea, South

Jihene Lachheb

Tunisia

Umberto Laforenza

Italy

Juan Jose Lahuerta

Spain

Majlinda Lako

United Kingdom

Aimee Landar

United States of America

Joseph Lane

United States of America

Simon Langdon

United Kingdom

Leopoldo Laricchia-Robbio

Spain

Pernille Lassen

Denmark

Najma Latif

United Kingdom

Lorenza Lazzari

Italy

Manuel Leal

Spain

Virna Leaner

South Africa

Jongho Lee

Korea, South

Mong Hong Lee

United States of America

Asada Leelahavanichkul

Thailand

Willem Lems

Netherlands

Nikoletta Lendvai

United States of America

Laura Lentini

Italy

Georg Lenz

Germany

Gregory Lesinski

United States of America

Pei-Feng Li

China

Jing Li

United States of America

Jinjun Li

China

Jun Li

China

Min Li

United States of America

Rui Li

China

Giovanni Li Volti

Italy

Han Liang

China

Evi Lianidou

Greece

Michael Liebman

United States of America

Yang Lifang

China

Liu Lijiang

China

Jizhen Lin

United States of America

Konghua Lin

Japan

Sheng-Xiang Lin

Canada

Shi-Ming Lin

Taiwan

Yi Lin

United States of America

Zhao Lin

United States of America

Lisa Lindesmith

Afghanistan

Wenhua Ling

China

Alexander Link

Germany

Daniela Lisini

Italy

Chun-Fu Liu

China

De-Pei Liu

China

Hongmin Liu

United Kingdom

Lei Liu

China

Mingyao Liu

Canada

Qi Liu

United States of America

Qi-Fa Liu

China

Ran Liu

China

Rong Liu

China

Wanli Liu

China

Yan Liu

China

Yu-Ming Liu

Taiwan

Markus W. Löffler

Germany

A. Inkeri Lokki

Finland

Lucia Lopalco

Italy

Elena Lopez

Spain

Jose Antonio Lopez-Guerrero

Spain

Frazer Lowe

United Kingdom

Bin Lu

China

Hua Lu

United States of America

Jiamiao Lu

United States of America

Pei-Jung Lu

Taiwan

Qun Lu

United States of America

Shixin Lu

China

Tracy Luckhardt

United States of America

Cora Lueders

Germany

Alessandro Lugli

Switzerland

Vivian Lui

United States of America

David Lum

United States of America

Christina Lund

Sweden

Troy Lund

United States of America

Maria Lung

Hong Kong

Per Johan Luthman

United States of America

Ming-Chieh Ma

Taiwan

Stephanie Ma

Hong Kong

Xinliang Ma

United States of America

Michael Maes

Thailand

Davide Maggi

Italy

Jared Magnani

United States of America

Mitra Mahdavi-Mazdeh

Iran

John Maher

United Kingdom

Toivo Maimets

Estonia

Arindam Maitra

India

Ajay Maker

United States of America

James Maloney

United States of America

Kwan Man

China

Mario Mandala

Italy

Masoud Manjili

United States of America

Maurilio Marcacci

Italy

Arivusudar Marimuthu

India

Jose Marin

Spain

Francesco Marincola

United States of America

Dimitra Marioli

Greece

Athina Markou

United States of America

Svetomir Markovic

United States of America

Eva Martinez-Balibrea

Spain

Yoshinori Marunaka

Japan

Giuseppe Masucci

Sweden

António Mata

Portugal

Suresh Mathivanan

Australia

Scot Matkovich

United States of America

Hisahiro Matsubara

Japan

Yutaka Matsuda

Japan

Michael A. Matthay

United States of America

Nicola Maurea

Italy

Joan Maurel

Spain

Hani Mawardi

United States of America

Ryan Mays

United States of America

J Philip Mccoy

United States of America

Brent Mccright

United States of America

Kenneth Mcgaffin

United States of America

Tara Mcmorrow

Ireland

Robert Medcalf

Australia

Shikhar Mehrotra

United States of America

Nehal Mehta

United States of America

Ignacio Melero

Spain

David Melodelima

France

Xianmei Meng

United States of America

Tiziana Meschi

Italy

Clement Meseda

United States of America

James Mezhir

United States of America

Lindsey Miles

United States of America

Wilson Miller Jr.

Canada

Rowan Milner

United States of America

Maria Chiara Mimmi

Italy

Wei-Ping Min

Canada

Boris Minev

United States of America

Salvatore Minisola

Italy

David Minor

United States of America

Laura Mitrofan

France

Christopher Mizer

United States of America

Hiroshi Mizuno

Japan

Simone Mocellin

Italy

Ralph Mocikat

Germany

Rajesh Mohanraj

United Arab Emirates

Lenny Moise

United States of America

Myth Mok

Australia

Jan Mol

Netherlands

Miguel-Angel Molina-Vila

Spain

Carlomaurizio Montecucco

Italy

Augusto Montezano

United Kingdom

Julia Montgomery

Canada

Jan-Renier Moonen

Netherlands

Ronald Moore

Canada

Violaine Moreau

France

David Morris

Australia

Pavlos Msaouel

United States of America

Hong-Hua Mu

United States of America

Brigitte Mueller-Hilke

Germany

Sumanta Mukherjee

United States of America

Hans Werner Müller

Germany

Elizabeth Mumper

United States of America

Sachiko Murase

United States of America

Antonino Musolino

Italy

Ioannis Mylonas

Germany

Alessio Naccarati

Italy

Cristina Nadal

Spain

Silvio Nadalin

Germany

Jayasree Nair

United States of America

Noriaki Nakai

Japan

Nobuhiro Nakamoto

Japan

Tetsuya Nakatsura

Japan

Eiichi Nakayama

Japan

Jeong-Seok Nam

Korea, South

Kaavya Narasimhalu

Singapore

Urs Nater

Germany

Shoji Natsugoe

Japan

Frank Naya

United States of America

Sadaf Naz

Pakistan

Armando Negri

Argentina

Brad Nelson

Canada

William Nelson

United States of America

Dirk M. Nettelbeck

Germany

Manish Neupane

United States of America

Daniel Neureiter

Austria

Ellen Neven

Belgium

Brian Neville

United Kingdom

Akira Nifuji

Japan

Jan Nilsson

Sweden

Torbjörn Nilsson

Sweden

Katharina Nimptsch

Germany

Tao Ning

China

Paola Nistico

Italy

Christopher Niyibizi

United States of America

Joseph Nkolola

United States of America

Constance Noguchi

United States of America

Songmi Noh

Korea, South

Keith Nolop

United States of America

Shigeaki Nonoyama

Japan

Giuseppe Danilo Norata

Italy

Tarcisio Not

Italy

Takahiro Ochiya

Japan

Matthias Ocker

Germany

José-Enrique O'connor

Spain

Norma O'donovan

Ireland

Paul Ogburn

Qatar

Keiichi Ohshima

Japan

Jeanne Oiticica

Brazil

Seiichi Okabe

Japan

Hideho Okada

United States of America

Daniel Olive

France

Oluwadayo Oluwadara

United States of America

Frank Ondrey

United States of America

Alexander Opotowsky

United States of America

Alexander Opotowsky

Aruba

Ester Orlandi

Italy

Pablo Ortega-Deballon

France

Brenna Osborne

Australia

Nurhan Ozlu

Turkey

Frederic Padilla

France

Debnath Pal

India

Peter Palese

United States of America

Nicolas Pallet

France

Giuseppe Palmieri

Italy

Nicola Palmieri

Italy

Rogelio J Palomino-Morales

Spain

Massimo Pancione

Italy

Gopal Pande

India

Hardev Pandha

United Kingdom

Rungnapa Pankla Sranujit

Thailand

Constantinos Pantos

Greece

Antonio Paoli

Italy

Laszlo Papp

United Kingdom

Luther Lalit Kumar Parameswaran Grace

Sweden

Vincenzo Parisi

Italy

Maura Parker

United States of America

Giorgio Parmiani

Italy

George Patrinos

Greece

Juan D. Pedrera-Zamorano

Spain

Ying Peng

China

Yu-Zhu Peng

China

Jeroen Pennings

Netherlands

Kristina Penniston

United States of America

Aleth Perdriger

France

Rolando Perez

Cuba

Alessandro Perrella

Italy

Lars J Petersen

Denmark

Ulrich Pfeffer

Italy

Mark Pickard

United Kingdom

Peter Pickkers

Netherlands

Gabriella Pietra

Italy

Martina Pigazzi

Italy

Alessandro Pileri

Italy

Asha Pillai

United States of America

Beena Pillai

India

Shari Pilon-Thomas

United States of America

Jef Pinxteren

Belgium

Maria Pia Pistillo

Italy

Martin Playford

United States of America

Nicole Pohl

United States of America

Waldemar Popik

United States of America

Laura Porretti

Italy

Giuseppe Portella

Italy

George Potamias

Greece

Jacques Pouyssegur

France

Thomas Povsic

United States of America

Sreenivasa Prasad

India

Enrico Proietti

Italy

Joseph Provost

United States of America

Grzegorz K. Przybylski

Poland

Peiyu Pu

China

Igor Puzanov

United States of America

Jie Qiao

China

Haibo Qiu

China

Peter Quesenberry

United States of America

Samuel Rabkin

United States of America

Lluigi Racioppi

United States of America

Anne Raimondo

United Kingdom

Mayur Ramesh

United States of America

Elena Ranieri

Italy

Heikki Rantala

Finland

Benedetta Raspini

Italy

Cyril Rauch

United Kingdom

Pierre-Emmanuel Rautou

France

Michael Reedijk

Canada

Jo-Anna Reems

United States of America

Abdul Rehman

Pakistan

Andrea Remo

Italy

Jiaqiang Ren

United States of America

Jia-Qiang Ren

United Kingdom

Pei-Gen Ren

China

Tao Ren

China

Xiaoping Ren

United States of America

Domenico Rendina

Italy

Yuan R-H

Taiwan

Domenico Ribatti

Italy

Maria Manuela Rigano

Italy

Renata Riha

United Kingdom

David Rimm

United States of America

Joseph Riss

United States of America

Martin Rivas

Spain

Tadeusz Robak

Poland

Jason Roberts

United States of America

Lewis Roberts

United States of America

Margo Roberts

United States of America

Paul Roberts

United States of America

Aldo Roccaro

United States of America

Rafael Rocha

Brazil

Allen Rodgers

South Africa

Luis Rodrigo

Spain

Leonardo Roever

Brazil

Joseph Rogers

United States of America

Richard Rokyta

Czech Republic

Jana Rolff

Germany

Guanghua Rong

China

Shawn Rose

United States of America

Rafael Rosell

Spain

Michael Rosenblum

United States of America

Yvonne Rosenstein

Mexico

Reema Roshan

India

Giulio Rossi

Italy

Violeta Rus

United States of America

Catherine Rush

Australia

Rasna Sabharwal

United States of America

Norihisa Saeki

Japan

Pål Sætrom

Norway

Zhi Xin Sahn

China

Khashayar Sakhaee

United States of America

Seema Saksena

United States of America

Takashi Saku

Japan

Antonio Salgado

Portugal

Michael Salman

Canada

Labidi-Galy Sana Intidhar

France

Linda Sandell

United States of America

William Sanders

United States of America

Randeep Sangha

Canada

Dario Sangiolo

Italy

Daniele Santini

Italy

Laura Sanz

Spain

Zoltan Sapi

Hungary

Anna Sapone

Italy

Devanand Sarkar

United States of America

Seetharama Sastry Konduru

Qatar

Noriyuki Sato

Japan

Yukie Sato-Kuwabara

Brazil

John Sayer

United Kingdom

Chris Scarlett

Australia

Francesco Paolo Schena

Italy

Dolores J. Schendel

Germany

Gabriella Schiera

Italy

Ingolf Schimke

Germany

Henrik Schmidt

Denmark

Roland Schmitt

Sweden

Jonathan Schneck

United States of America

Joachim Schneider

Germany

Reinhard Schneider

Luxembourg

Stephen Schneider

United States of America

Ralf Schubert

Germany

Hanneke Schuitemaker

Netherlands

Gerold Schuler

Germany

Hildegard M. Schuller

United States of America

Joachim Schultze

Germany

Albrecht Schwab

Germany

Alfredo Scillitani

Italy

Michael Seaman

United States of America

Werner Seeger

Germany

Ludovica Segat

Italy

Kim Selting

United States of America

Patricia Semedo

Brazil

Nilkantha Sen

United States of America

Shantanu Sengupta

India

Mukund Seshadri

United States of America

Alex Sgambato

Italy

Rachana Shah

United States of America

Aasef Shaikh

United States of America

Jingxuan Shan

Qatar

Anil Shanker

United States of America

Thomas Shanley

United States of America

Arulkumaran Shanmugam

United States of America

Mukut Sharma

United States of America

R Shaw

United Kingdom

Qing-Bai She

United States of America

Farah Sheikh

United States of America

Bairong Shen

China

Chia-Rui Shen

Taiwan

Lizong Shen

China

Xian Shen

China

Xizhong Shen

China

Wang Sheng

China

Sung Joon Shin

Korea, South

Ken Shinmura

Japan

Atsushi Shiozaki

Japan

Alex Shortt

United Kingdom

Imad Shureiqi

United States of America

Jason Sicklick

United States of America

Roswitha Siener

Germany

Andre R. Simon

United Kingdom

Josko Sindik

Croatia

Pankaj Singh

United States of America

Bruno Sinn

Germany

Sandhya Sitasawad

India

Cristina Skrypnyk

Bahrain

Roman Skulec

Czech Republic

John Sleasman

United States of America

David Smadja

France

Evelien Smits

Belgium

Elke Sokoya

Australia

Libing Song

China

Lijun Song

China

Richie Soong

Singapore

Hermona Soreq

Israel

Federica Sotgia

United Kingdom

Claudio Spada

Italy

Giulio C. Spagnoli

Switzerland

Paul Span

Netherlands

Bart Spee

Netherlands

Claude Sportes

United States of America

Donatella Spotti

Italy

Thilo Sprenger

Germany

Paul Squires

United Kingdom

Geetha Srikrishna

United States of America

Giovanni Stallone

Italy

Maria Stamelou

Greece

Norbert Stefan

Germany

Ulrike Stein

Germany

R. Scott Stephens

United States of America

Christian Stock

Germany

Nikolas Hendrik Stoecklein

Germany

Katarzyna Stolarz-Skrzypek

Poland

Walter Storkus

United States of America

David Stroncek

United States of America

Jeppe Sturis

Denmark

Auguste Sturk

Netherlands

Changqing Su

China

Anthony Suffredini

United States of America

Kimberly Sulivan

United States of America

Baocun Sun

China

Hongye Sun

China

Hui-Chuan Sun

China

Wei Sun

China

Xueying Sun

New Zealand

Jian Suo

China

Anil Suri

India

Aladar Szalay

United States of America

Mario Sznol

United States of America

Elda Tagliabue

Italy

Pierosandro Tagliaferri

Italy

Chien-Kuo Tai

Taiwan

Masafumi Takahashi

Japan

Takashi Takata

Japan

Naokazu Takeda

Japan

Eng M Tan

United States of America

Hao Tang

China

Jun-Ming Tang

China

Kai-Fu Tang

China

Li Tang

China

Wei Tang

Japan

Hideki Tanzawa

Japan

Yongguang Tao

China

Seyed Khosrow Tayebati

Italy

Bin Tean Teh

Italy

Muy-Teck Teh

United Kingdom

Seagal Teitz-Tennenbaum

United States of America

Mustafa Tekin

United States of America

Grethe Tell

Norway

Masaki Terabe

United States of America

Wolfgang Thasler

Germany

Martin Thelin

Sweden

George Theodoropoulos

Greece

Christoph Thiemermann

United Kingdom

Ling Tian

China

Wen-Dong Tian

China

Hwei-Fang Tien

Taiwan

Takashi Tokino

Japan

Sara Tomei

Qatar

Jorge Toro

United States of America

Tomomi Toubai

United States of America

Cyril Touboul

France

Marek Treiman

Denmark

Giovanni Tripepi

Italy

Jakob Troppmair

Austria

Kwong Tsang

United States of America

Masahiko Tsujii

Japan

Masayuki Tsuneki

United States of America

Sandra Tuyaerts

Belgium

Lorenzo Uccellini

United States of America

Krishna Udayakumar

United States of America

Hirotsugu Uemura

Japan

Michae Uhlin

Sweden

MF Ullah

India

Masuko Ushio-Fukai

United States of America

Daniel Vallera

United States of America

Willem Jan Marie Van De Ven

Belgium

Peter Van Der Kraan

Netherlands

Martine Van Grotel

Netherlands

Inge Van Houdt

Netherlands

Frederik-Jan Van Schooten

Netherlands

Viggo Van Tendeloo

Belgium

Menno Van Zelm

Netherlands

Guido Vanham

Belgium

Ravi Varatharajalu

United States of America

Marileila Varella-Garcia

United States of America

Catalin Vasilescu

Romania

David Vaudry

France

Juergen Veeck

Germany

Vaneja Velenik

Slovenia

Pedro L Vera

United States of America

Laura Vera-Ramirez

Spain

Samet Verim

Turkey

Bhavna Verma

United States of America

Fabio Vescini

Italy

John Vetto

United States of America

Giuseppe Vezzoli

Italy

Fabien Vidal

France

Fernando Vidal-Vanaclocha

Spain

Natassia Vieira

United States of America

Jesus Villar

Spain

Elizabeth Vincan

Australia

Maria Luisa Visciano

Italy

Sowmya Viswanathan

Canada

Michele Vitacca

Italy

Massimo Vitale

Italy

Ioannis Voutsadakis

Canada

Premkumar Vummidi Giridhar

United States of America

Nathan Wall

United States of America

W Walther

Germany

Gang Wang

United States of America

Guanghu Wang

United States of America

Haibin Wang

China

Honglin Wang

China

Jian Wang

China

Mong-Heng Wang

United States of America

Shengdian Wang

China

Ting Wang

China

Wei Wang

China

Xiangdong Wang

China

Xuejun Wang

United States of America

Yaohe Wang

United Kingdom

Yingqun Wang

United States of America

Youzhen Wang

China

Yuan Wang

China

Zhou Wang

United States of America

Zhu Wangyu

China

Dawn Ward

United States of America

Lorraine Ware

United States of America

Jonathan Watson

United Kingdom

Sarath Wattegama

Sri Lanka

David Waugh

United Kingdom

David Webb

United States of America

Grzegorz Wegrzyn

Poland

Wei Wei

United States of America

George Weiner

United States of America

Thomas Weiss

Germany

Christine Wells

Australia

Zilong Wen

Hong Kong

Scott Wenderfer

United States of America

Honglei Weng

Germany

Jing-Ru Weng

Taiwan

Joao Pedro Werneck De Castro

Brazil

Meir Wetzler

United States of America

Stephen Whelan

United States of America

Britt Wildemann

Germany

David Williams

United States of America

David Williams

Ireland

Gabrielle Williams

Australia

Paul Wolters

United States of America

Hector Wong

United States of America

Andrea Worschech

Qatar

Mingfu Wu

United States of America

Tianfu Wu

United States of America

Bing Xia

China

Harry Xia

United States of America

Jinglin Xia

China

Wu Xiaodan

China

Li Xingyi

China

Qing Xiong

China

Meifeng Xu

United States of America

Rui-Hua Xu

China

Ying Xu

China

Jian Yan

China

Jun Yan

United States of America

Hiroaki Yanagimoto

Japan

An-Gang Yang

China

Dawei Yang

China

Hong Yang

United States of America

Jianjun Yang

China

Lily Yang

United States of America

Qingwu Yang

China

Zu-Guo Yang

China

Takahiro Yasui

Japan

Kosei Yasumoto

Japan

Li Ye

United States of America

Jim Yeung

United States of America

Yongping You

China

Shiyu Long Yu Long

China

Yongyi Yuan

China

Anna Zaczek

Poland

Maurizio Zanetti

United States of America

Paul Zarogoulidis

Greece

Fahd Zarrouf

United States of America

Yekaterina Zaytseva

United States of America

Jiping Zeng

China

Qingping Zeng

United States of America

Yi Zeng

China

Thorsten Zenz

Germany

Gianpaolo Zerbini

Italy

Dimitrios Zeugolis

Ireland

Guo-Jun Zhang

China

Lucy Zhang

United States of America

Xianglan Zhang

Korea, South

Xiao-Shi Zhang

China

Xu Dong Zhang

Australia

Yu Zhang

United States of America

Zengli Zhang

China

Zaorui Zhao

United States of America

Guoping Zheng

Australia

Min Zheng

China

Shu Zheng

China

Huijun Zhi

United Kingdom

Aimin Zhou

United States of America

Chuanlong Zhu

United States of America

Zhitu Zhu

China

Ewa Ziemann

Poland

Anna Linda Zignego

Italy

Massimo Zollo

Italy

Amina Zoubeidi

Canada

